# Evaluation of Human Leukocyte Antigen Class I and Class II in End-Stage Renal Disease Occurrence in Indonesian Transplantation Patients

**DOI:** 10.1155/2021/4219822

**Published:** 2021-10-11

**Authors:** Hani Susianti, Dwi Priyadi Djatmiko, I Komang Adi Widana, Deasy Ayuningtyas Tandio, Catur Suci Sutrisnani, Singgih Pudjo Wahono, Ira Puspitawati

**Affiliations:** ^1^Clinical Pathology Departement, Dr. Saiful Anwar General Hospital, Faculty of Medicine, Universitas Brawijaya, Malang, Indonesia; ^2^Clinical Pathology Departement, Dr. Sardjito General Hospital, Faculty of Medicine, Public Health and Nursing, Universitas Gadjah Mada, Yogyakarta, Indonesia

## Abstract

**Background:**

Genetic studies of end-stage renal disease (ESRD), including those of human leukocyte antigen (HLA) genes, have been reported in several populations but have not yet been evaluated in Indonesia. Some studies have reported that these genes had a substantial role in ESRD. This study aims to analyze the association between HLA genes and ESRD within the Indonesian community.

**Method:**

A retrospective study to investigate HLA class I and II alleles to find out the distribution of HLA-A, -B, -C, -DPB1, -DQB1, and -DRB1 in renal transplant recipients and to ascertain their role in susceptibility to ESRD was performed on totally 149 subjects, consisting of 69 ESRD patients and 80 healthy controls. HLA typing was determined using Luminex techniques. The allele and haplotype frequencies were compared between ESRD patients and controls.

**Result:**

High-frequency alleles were HLA-A*∗*24 (43.6%), B*∗*15 (38.2%), C*∗*08 (30.8%), DRB1*∗*12 (47.3%), DQB1*∗*03 (50.6%), and DPB1*∗*13 (22.5%). HLA-A*∗*24 (*p*=0.01) and HLA-B*∗*35 (*p*=0.02) were associated with a protective effect, with OR 0.537 (95%CI 0.34–0.86) and 0.316 (95%CI 0.11–0.88), respectively. There were some two-locus haplotypes associated with susceptibility to ESRD, such as B*∗*15-DRB1*∗*12, B*∗*13-DRB1*∗*15, A*∗*02-B*∗*15, A*∗*02-C*∗*08, and B*∗*13-DQB1*∗*05. HLA-A*∗*02-B*∗*15-DRB1*∗*12 and A*∗*24-B*∗*13-DRB1*∗*15 appear to be associated with susceptibility to ESRD.

**Conclusion:**

The allele groups of HLA-A*∗*24 and HLA-B*∗*35 are associated with protection from ESRD. Meanwhile, HLA-B*∗*13-DRB1*∗*15 and A*∗*24-B*∗*13-DRB1*∗*15 are the most frequent HLAs associated with ESRD in two-locus and three-locus haplotype, respectively.

## 1. Introduction

The incidence of ESRD in the world is increasing as well as in Indonesia. The Indonesian Renal Registry showed that ESRD incidence rose from 9649 in 2010 to 30831 in 2017. At the same time, the prevalence of ESRD increased from 11484 in 2010 to 77892 in 2017. The causes of kidney disease are mainly due to hypertension and diabetes mellitus, with the 1-year survival rate of 83%, while the 5-year survival rate was 51.9%. The leading cause of death in 37% of cases was cardiovascular disorders [1].

End-stage renal disease (ESRD) is suspected to be influenced by genetic and nongenetic factors. Many genetic factors cause ESRD, and human leukocyte antigen (HLA) is an essential factor. HLA is encoded by the major histocompatibility complex (MHC) located on chromosome 6p21.3. The HLA molecule binds and presents peptide to T lymphocytes in cell-mediated immune response and plays a crucial role in shaping the T-cell repertoire and is also associated with allograft rejection [2, 3]. The correlation of HLA polymorphism with ESRD can be caused by HLA association with the aetiology and the progression of kidney disease. For example, in a Mexican population with type 2 diabetes mellitus (T2DM), HLA-DRB1*∗*15:02 is associated with diabetic kidney disease. Meanwhile, specific HLA can also trigger a profibrogenic T-cell phenotype, possibly contributing to the progression of kidney disease severity [3]. Understanding the genetic factors that influence kidney disease is essential to help estimate the prognosis and prevention of ESRD.

Successful transplantations correlate with the HLA compatibility level between recipients and donors. Renal allotransplantation improves the quality of life and increases survival in patients with ESRD compared with long-term dialysis treatment. Primary renal allograft loss is associated with significant mortality. Therefore, the information about frequencies of HLA haplotype occurs in a population, and the association between HLA and ESRD in the Indonesian community is important. In this study, the distributions of HLA-A, HLA-B, HLA-C, HLA-DPB1, HLA-DQB1, and HLA-DRB1 in the renal transplant recipients and donors are obtained to determine HLA antigen frequencies.

## 2. Methods

### 2.1. Population Samples

This study was observational and retrospective. The data were collected from the biomolecular laboratory databases of Dr. Saiful Anwar General Hospital from 2017 to 2020. We investigated HLA class I and II alleles to determine the distribution of HLA-A, HLA-B, HLA-C, HLA-DPB1, HLA-DQB1, and HLA-DRB1 and to ascertain their role in susceptibility to ESRD in patients considering renal transplantation and healthy controls. The inclusion criteria were the subjects had complete HLA typing data from 6 loci, not family related, and Indonesian population.

### 2.2. DNA Extraction

DNA was isolated from whole blood containing Acid Citrate Dextrose (ACD) solution, using a Genomic DNA Isolation kit, according to the manufacturer's instructions (Qiagen). In brief, 20 *μ*L of Qiagen protease and 200 *μ*L of whole blood were added into a 1.5 mL microcentrifuge tube. 200 *μ*L of buffer (AL) was added and then incubated at 56°C for 10 minutes, and 200 *μ*L of ethanol (100%) was added and centrifuged. The mixture was transferred into a QIAamp mini spin column, the tube was centrifuged, the QIAamp mini spin column was placed in a clean 2 mL collection tube, and the tube containing the filtrate was discarded. 500 *μ*L of buffer (AW1) was added and centrifuged, then the QIAamp mini spin column was placed in a clean 2 mL collection tube, and the tube containing the filtrate was discarded. The procedure was repeated with 500 *μ*L of buffer (AW2), and then, the QIAamp mini spin column was placeed in a clean 1.5 mL microcentrifuge tube. Then, 200 *μ*L of elusion buffer (AE) was added and centrifuged, the QIAamp mini spin column was discarded, and the microcentrifuge tube containing the eluted DNA was stored at −80°C.

### 2.3. DNA Typing

HLA typing of loci HLA-A, HLA-B HLA -C, HLA-DPB1, HLA-DQB1, and HLA-DRB1 was performed by Luminex Labscan 100 using the sequence-specific oligonucleotide probes (SSO) method. In summary, 2 *μ*L of the target DNA is amplified using a group-specific primer. The PCR product is denatured and allowed to rehybridize to complementary DNA probes conjugated to fluorescently coded microspheres. A flow analyzer, the LABScan™ 100, identified the fluorescent intensity of PE (phycoerythrin) on each microsphere. The HLA Fusion software (ONE LAMBDA, INC, USA) will analyze the HLA typing for allele identification based on the reaction pattern compared to patterns associated with published HLA gene sequences.

### 2.4. Statistical Analysis

HLA allele, two-locus, and three-locus haplotype frequencies were performed by direct counting in percentage. Two-locus haplotype frequencies in this study will be compared with the two-locus haplotype from the other population because more studies reported the two-locus haplotype than the three-locus haplotype. However, the three-locus haplotype (HLA-ABDR) was considered more relevant in this regard, so we also performed the three-locus haplotype in this study. The association between HLA allele with ESRD was estimated with Odds Ratio (OR) and 95% confident intervals, with *p* < 0.05 was considered statistically significant. Odds Ratio (OR) value greater than one was deemed positive or susceptible to ESRD, meanwhile OR values less than one were considered protective to ESRD. The exact test was used to evaluate the assumption of Hardy–Weinberg Equilibrium (HWE). The software for statistical analysis was the SPSS version 23 and OpenEpi. The institutional ethics board of Dr. Saiful Anwar General Hospital has approved the study (ethical number 400/141/K.3/302/2020).

## 3. Results

### 3.1. Characteristics of Study Participants and Hardy–Weinberg Equilibrium Tests

**Figure 1:**
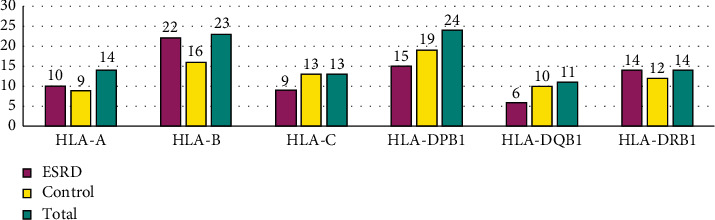
Numbers of HLA-A, HLA-B, HLA-C, HLA-DPB1, HLA-DQB1, and HLA-DRB1 allele groups in ESRD patients, control, and both groups.

The study collected HLA data from 81 ESRD and 87 control participants. According to the inclusion criteria, 69 ESRD patients and 80 donors, a total of 149 subjects, were selected. There was a male predominance among the control group (63.8%) and ESRD group (51.3%). The allele number of HLA-A, HLA-B, HLA-C, HLA-DPB1, HLA-DQB1, and HLA-DRB1 in ESRD patients and donors are summarized in column charts shown in Figure 1. The HWE tests of the HLA-A, -B, -C, -DRB1, -DQB1, and -DRP1 loci showed *p* > 0.05.

### 3.2. Allele Frequencies at HLA-A, HLA-B, HLA-C, HLA-DPB1, HLA-DQB1, and HLA-DRB1 Loci in Renal Transplant Recipients and Donors

The high-frequency alleles of HLA class I was HLA-A*∗*24 (35.50% in ESRD,50.60% in control group, and 43.60% in both groups), HLA-B*∗*15 (37.70% in ESRD, 38.20% in control group, and 38.20% in both groups), and HLA-C*∗*08 (31.20% in ESRD, 30% in control group, and 30.80% in both groups).  Table 1 shows the class I HLA types with frequency >1% in the ESRD or control groups. If the frequency is 1% or less, the data were not performed. According to Table 1, HLA-A*∗*24 (*p*=0.01) and HLA-B*∗*35 (*p*=0.02) were associated with a protective effect, with OR 0.537 (95%CI 0.34–0.86) and 0.316 (95%CI 0.11–0.88), respectively.

Table 2 indicates the class II HLA types with frequency >1% in the ESRD or control groups. The high-frequency alleles of HLA class II were HLA-DRB1*∗*12 (44.20% in ESRD, 50% in control group, and 47.30% in both groups), HLA-DQB1*∗*03 (49.30% in ESRD, 51.90% in control group, and 50.6% in both groups), and HLA-DPB1*∗*13 (22.50% in ESRD, 22.50% in control group, and 22.5% in both groups). HLA class II was not associated with ESRD.

Table 3 shows the association of two-locus haplotypes with ESRD with a significant odds ratio between the two groups (*p* value <0.05). There are some haplotypes associated with susceptibility to ESRD, namely, B*∗*15-DRB1*∗*12, B*∗*13-DRB1*∗*15, A*∗*02-B*∗*15, A*∗*02-C*∗*08, and B*∗*13- DQB1*∗*05. Meanwhile, the other haplotypes are associated with protection from ESRD.

Table 4 indicates the association of the three-locus haplotypes with ESRD with a significant odds ratio between the two groups (*p* value <0.05). Two haplotypes are associated with susceptibility to ESRD, where the highest OR is HLA-A*∗*24-B*∗*13-DRB1*∗*15 (OR = 8.171), followed by HLA-A*∗*02-B*∗*15-DRB1*∗*12 (OR = 5.724). The haplotype of HLA-A*∗*24-B*∗*35-DRB1*∗*12 and HLA-A*∗*24-B*∗*35-DRB1*∗*15 is negatively associated with ESRD.

## 4. Discussion

Aside from its essential role in determining donor-recipient immune compatibility in organ transplantation, HLA genotyping is performed routinely as part of the diagnostic work-up of certain autoimmune diseases. The HLA genotyping also has contributed to understanding several diseases' pathogenesis, including ESRD [3].

Our study observed that the ESRD participants consisted of 51.3% males and 48.7% females. It is similar to the research by Panigrahi et al. [4] and Tuladhar et al. [5], which reported that most ESRD patients were males.

The data of HLA alleles in our study showed heterogeneity in both HLA class I or class II. The number of HLA-B alleles is higher than HLA-A and HLA-C in HLA class 1. Meanwhile, HLA class II indicated that the number of HLA-DPB1 alleles is higher than HLA-DR and HLA-DQB1. The heterogeneity of HLA alleles results in amino acid substitutions that predominantly involve peptide binding sites for the effective display of a broad peptide to CD4+ and CD8+ T cells. As of July 2018, the polymorphisms of HLA-A, HLA-B, and HLA-C had 9,341 different proteins and 5,355 proteins in the polymorphisms of HLA-DR, HLA-DQ, and HLA-DP [3].

The high frequency of HLA-A*∗*24 in our study is also observed similarly in South Indian, northern Indian, and Bangladeshi Bengali populations [5–7]. The frequent alleles such as HLA-A*∗*02 and A*∗*11 were also observed by Ali et al., and this HLA correlated with the Bangladeshi Bengali population. The most common alleles in Nepal, such as HLA-B*∗*15 and DRB1*∗*12 [5], were also found in high frequencies in our study. Pradana et al. stated that the Indonesian HLA profile had high variation at HLA-DQA1*∗*06:01, DQB1*∗*03:01, DRB1*∗*12:02, A*∗*24:07, B*∗*15:02, B*∗*75, and B*∗*18:02 [8]. These data are consistent with our research that found the high frequency of HLA-A*∗*24, B*∗*15, DRB1*∗*12, and DQB1*∗*03. Polymorphism in the HLA system is also used as a tool for anthropological studies. Genetic distances and correspondence analysis demonstrated that the allele and haplotype distribution of class I and class II loci are racially and geographically restricted [9, 10].

HLA-A*∗*24 had a negative correlation with ESRD, according to our data. However, Cao et al. reported that HLA-A*∗*24 in ESRD patients is significantly higher than in the controls and associated with ESRD in the Cantonese population [11]. The different result of the study with our study is caused by that the HLA expression that affects ESRD may be influenced by not only one locus but also many loci of HLA. For example, according to our study, HLA-A*∗*24-B*∗*13-DRB1*∗*15 is strongly associated with ESRD susceptibility. So, the role of HLA-A*∗*24 in chronic kidney disease and ESRD still needs more study in the future because the HLA system plays a pivotal role in the antigen presentation of intracellular and extracellular peptides and the regulation of innate and adaptive immune responses. Considering the ability of HLA to influence the thymic selection and peripheral energy of T cells, its role in the pathogenesis of kidney disease needs to be investigated [12].

HLA-B*∗*35 had a negative association with ESRD based on our study. However, in Brazil, the research by Ravazzi-Gauch et al. stated that the most common alleles for each locus among ESRD or renal transplantation patients were HLA-A*∗*02, B*∗*35, and DRB*∗*11 [13]. In China, the HLA-B locus showed HLA-B*∗*15:01, B*∗*55:02, and B*∗*39:05 emerged as susceptible alleles, whereas no protective allele was found [14]. Meanwhile, in our study, the primary HLA-B allele in the kidney transplant patient group was HLA-B*∗*15. In the HLA system, allele and haplotype distribution differ between various ethnic groups or between the members of the same ethnic group living in different geographic areas, as shown in previous studies [14, 15].

HLA-C, HLA-DR, HLA-DQ-B1, and HLA-DPB1 did not associate with ESRD, according to our data. This result is different from the study by Lowe et al. that reported HLA association with kidney function on 401,307 white British subjects. They showed that 22 types of HLA were associated with increased eGFR and 11 types of HLA were associated with decreased eGFR. As many as 7 HLA out of 11 types are associated with CKD or ESRD, namely, HLA-A*∗*01:01, B*∗*08:01, C*∗*07:01, DRB1*∗*03:01, DRB3*∗*01:01, DQA1*∗*05:01, and DQB1*∗*02:01 [16]. In the ESRD group, our study indicated that the most HLA-DPB1 locus was HLA-DPB1*∗*13, but there was no significant difference with the control group. The association between HLA-DPB1 and ESRD has never been reported in Indonesia, but some studies in Indonesia stated that HLA-DPB1 is associated with hepatitis B infection susceptibility [17–19].

Some two-locus haplotypes were associated with susceptibility to ESRD in our study, such as HLA-A*∗*02-B*∗*15, A*∗*02-C*∗*08, B*∗*15-DRB1*∗*12, B*∗*13-DRB1*∗*15, and B*∗*13-DQB1*∗*05. This result is different from the research by Hamdi that reported the association of two-locus haplotypes HLA-A*∗*01-DRB1*∗*13 and HLA-A*∗*30-DRBI*∗*03 with ESRD in Saudi Arabian population [20]. In Venezuela, a study by Rivera et al. stated that the haplotypes positively associated with ESRD were HLA-A*∗*02-B*∗*51, HLA-A*∗*02-B*∗*53, HLA-A*∗*23-B*∗*38, and HLA-A*∗*68-B*∗*38 [21].

According to our data, HLA-A*∗*02-B*∗*15-DRB1*∗*12 and HLA-A*∗*24-B*∗*13-DRB1*∗*15 were three-locus haplotypes associated with susceptibility to ESRD. HLA-A*∗*24-B*∗*13-DRB1*∗*15 had a strong association with ESRD with OR 8.171. Nevertheless, the study by Cao et al. in the Cantonese community, a representative southern population of China, showed that HLA-A*∗*11-B*∗*27-DRB1*∗*04 in ESRD patients is significantly higher than that in the controls [11].

Based on the data of HLA and ESRD given above, there are different results in different studies and populations. This difference may be influenced by the disease that causes kidney abnormalities. HLA has substantial risk factors in most immune-mediated renal disorders. Together with other genetic and environmental factors, HLA causes loss of tolerance and autoimmune-mediated inflammation of the kidney [3, 22]. For example, Karnes et al. reported that HLA-DRB1*∗*04 and HLA-DQB1*∗*03:02 had an association with diabetic kidney disease. The association of HLA-DQB1*∗*03:02 and ESRD or kidney transplantation was weak (OR = 1.4), but this HLA had the risk of type 1 diabetes and diabetic kidney disease with OR 7.1 [23].

The limitations of our study were the small number of ESRD and healthy control participants. Further research with a more significant number of samples is needed. However, our research is expected to give a piece of information on the association between HLA and ESRD, especially in Indonesia, thus allowing the management of chronic kidney disease patients with HLA susceptibility more precisely and effectively.

## 5. Conclusions

The result of this study showed heterogeneity in both HLA class I or class II antigens, and some HLA polymorphisms have an association with ESRD in the Indonesian population. HLA-A*∗*24 and HLA-B*∗*35 are associated with protection from ESRD, whereas HLA-B*∗*13-DRB1*∗*15 is the most frequent HLA related to susceptibility to ESRD in the two-locus haplotype and HLA-A*∗*24-B*∗*13-DRB1*∗*15 in the three-locus haplotype.

## Figures and Tables

**Table 1:** HLA class I allele frequency.

HLA subtype	Alleles	ESRD		Control		OR	OR 95% CI	*p* value
		*n*	%	*n*	%			
HLA-A	A*∗*01	0	0	2	1.30	—	—	—
	A*∗*02	31	22.50	23	14.40	1.726	0.95–3.13	0.10
	A*∗*11	34	24.60	33	20.60	1.258	0.73–2.17	0.49
	A*∗*24	49	35.50	81	50.60	0.537	0.34–0.86	0.01^*∗*^
	A*∗*29	2	1.40	0	0	—	—	—
	A*∗*31	2	1.40	0	0	—	—	—
	A*∗*33	9	6.50	13	8.10	0.789	0.33–1.91	0.76
	A*∗*34	6	4.30	5	3.10	1.409	0.42–4.72	0.80
	A*∗*74	3	2.20	0	0	—	—	—

HLA-B	B*∗*07	4	2.90	4	2.50	1.164	0.29–4.74	1
	B*∗*08	2	1.40	0	0	—	—	—
	B*∗*13	7	5.10	3	1.90	2.796	0.71–11.03	0.23
	B*∗*15	52	37.70	62	38.80	0.956	0.60–1.53	0.94
	B*∗*18	15	10.90	16	10	1.098	0.52–2.31	0.95
	B*∗*27	8	5.80	8	5	1.169	0.43–3.20	0.96
	B*∗*35	5	3.60	17	10.60	0.316	0.11–0.88	0.02^*∗*^
	B*∗*38	8	5.80	11	6.90	0.834	0.33–2.14	0.89
	B*∗*39	4	2.90	2	1.30	2.358	0.43–13.08	0.55
	B*∗*40	8	5.80	8	5	1.169	0.43–3.20	0.96
	B*∗*44	4	2.90	12	7.50	0.368	0.12–1.17	0.13
	B*∗*51	7	5.10	11	6.90	0.724	0.27–1.92	0.69
	B*∗*52	1	0.70	2	1.30	0.577	0.05–6.43	1
	B*∗*56	3	2.20	2	1.30	1.756	0.29–10.66	0.86
	B*∗*58	3	2.20	1	0.60	3.533	0.36–34.36	0.52

HLA-C	C*∗*01	6	4.30	2	1.20	3.977	0.69–51.79	0.15
	C*∗*03	14	10.10	13	8.10	1.277	0.58–2.82	0.68
	C*∗*04	20	14.50	29	18.10	0.735	0.40–1.36	0.41
	C*∗*06	4	2.20	3	1.90	1.163	0.23–5.86	1
	C*∗*07	40	29.70	50	31.30	0.93	0.57–1.53	0.87
	C*∗*08	44	31.20	48	30	1.056	0.64–1.73	0.93
	C*∗*12	2	0.40	2	1.30	1.162	0.16–8.36	1
	C*∗*14	5	3.60	9	5.60	0.631	0.21–1.93	0.59
	C*∗*15	5	3.60	3	1.90	1.967	0.46–8.39	0.57

^*∗*^ *p* < 0.05. HLA types with frequency >1% were performed in ESRD or control groups. HLA: human leukocyte antigen; ESRD: end-stage renal disease.

**Table 2:** HLA class II allele frequency.

HLA subtype	Alleles	ESRD		Control		OR	OR 95% CI	*p* value
		*n*	%	*n*	%			
HLA-DRB1	DRB1*∗*03	5	3.60	2	1.30	2.97	0.567–15.56	0.34
	DRB1*∗*04	6	4.30	7	4.40	0.994	0.326–3.03	1
	DRB1*∗*07	9	6.50	14	8.80	0.728	0.305–1.74	0.62
	DRB1*∗*08	7	5.10	4	2.50	2.084	0.597–7.28	0.39
	DRB1*∗*09	4	2.90	3	1.90	1.562	0.344–7.10	0.84
	DRB1*∗*10	3	2.20	0	0	—	—	—
	DRB1*∗*11	3	2.20	4	2.50	0.867	0.191–3.94	1
	DRB1*∗*12	61	44.20	80	50	0.792	0.502–1.25	0.38
	DRB1*∗*14	5	3.60	3	1.90	1.967	0.462–8.39	0.57
	DRB1*∗*15	31	22.50	38	23.80	0.93	0.542–1.60	0.90
	DRB1*∗*16	2	1.40	4	2.50	0.574	0.103–3.18	0.83

HLA-DQB1	DQB1*∗*02	11	8	17	10.70	0.729	0.329–1.61	0.56
	DQB1*∗*03	68	49.30	83	51.90	0.901	0.572–1.42	0.74
	DQB1*∗*04	5	3.60	6	3.70	0.965	0.288–3.23	1
	DQB1*∗*05	33	23.90	31	19.30	1.308	0.752–2.28	0.42
	DQB1*∗*06	21	15.20	23	14.40	1.069	0.563–2.03	0.97

HLA-DPB1	DPB1*∗*01	5	3.60	6	3.80	0.965	0.288–3.23	1.00
	DPB1*∗*02	20	14.50	13	8.10	1.917	0.915–4.01	0.12
	DPB1*∗*03	8	5.80	15	9.40	0.595	0.244–1.45	0.35
	DPB1*∗*04	29	21	34	21.30	0.986	0.564–1.72	1.00
	DPB1*∗*05	24	17.40	26	16.30	1.085	0.591–1.99	0.91
	DPB1*∗*13	31	22.50	36	22.50	0.998	0.578–1.72	1.00
	DPB1*∗*14	0	0	4	2.50	—	—	—
	DPB1*∗*19	1	0.70	2	1.30	0.577	0.052–6.43	1
	DPB1*∗*26	0	0	2	1.30	—	—	—
	DPB1*∗*31	0	0	2	1.30	—	—	—
	DPB1*∗*105	4	2.90	2	1.30	2.358	0.425–13.08	0.55
	DPB1*∗*296	7	5.10	10	6.30	0.802	0.297–2.17	0.86
	DPB1*∗*350	3	2.20	2	1.30	1.756	0.289–10.66	0.86

HLA types with frequency >1% were performed in ESRD or control groups. HLA: human leukocyte antigen; ESRD: end-stage renal disease.

**Table 3:** Association of two-locus haplotypes with ESRD.

	Haplotypes	ESRD		Control		*p* value	OR	OR 95%CI
		*n*	%	*n*	%			
HLA-A and HLA-B	A*∗*02-B*∗*15	26	9.42	14	2.19	0.022	2.273	1.162–4.446
	A*∗*24*-*B*∗*35	5	1.81	24	3.59	0.002	0.238	0.089–0.635
	A*∗*24-B*∗*15	35	12.68	65	10.16	0.016	0.569	0.364–0.890

HLA-A and HLA-C	A*∗*02-C*∗*08	22	7.97	10	1.56	0.014	2.685	1.249–5.774
	A*∗*24-C*∗*08	27	9.78	53	8.28	0.020	0.546	0.333–0.896

HLA-A and HLA-DRB1	A*∗*24-DRB1*∗*12	51	18.48	87	13.59	0.015	0.607	0.410–0.897

HLA-A and HLA-DQB1	A*∗*24-DQB1*∗*03	53	19.20	85	13.28	0.042	0.657	0.445–0.969

HLA-B and HLA-C	B*∗*35-C*∗*04	5	1.81	24	3.75	0.001	0.228	0.086–0.605

HLA-B and HLA-DRB1	B*∗*15-DRB1*∗*12	61	22.10	39	6.09	0.001	2.044	1.317–3.172
	B*∗*13-DRB1*∗*15	9	3.26	1	0.16	0.010	10.75	1.354–85.410
	B*∗*35-DRB1*∗*15	1	0.36	9	1.41	0.036	0.125	0.015–0.998
	B*∗*35-DRB1*∗*12	5	1.81	17	2.66	0.037	0.328	0.119–0.903

HLA-A and HLA-DQB1	B*∗*35-DQB1*∗*03	5	1.81	17	2.66	0.037	0.329	0.120–0.903
	B*∗*13-DQB1*∗*05	8	2.90	1	0.16	0.021	9.522	1.184–76.610

HLA: human leukocyte antigen; ESRD: end-stage renal disease.

**Table 4:** Association of three-locus haplotypes with ESRD.

	ESRD		Control		*p* value	OR	OR 95%CI
	*n*	%	*n*	%			
A*∗*02-B*∗*15-DRB1*∗*12	33	5.98	7	1.01	0.001	5.724	2.512–13.050
A*∗*24-B*∗*35-DRB1*∗*12	7	1.27	34	5.34	0.001	0.227	0.100–0.518
A*∗*24-B*∗*35-DRB1*∗*15	1	0.18	9	1.41	0.036	0.126	0.016–1.003
A*∗*24-B*∗*13-DRB1*∗*15	7	1.27	1	0.15	0.043	8.171	1.002–66.610

HLA: human leukocyte antigen; ESRD: end-stage renal disease.

## Data Availability

The database for the study can be acquired from the principal investigator, Hani Susianti, hanisusianti.fk@ub.ac.id.

## References

[1] Abdurahman A., Bandiara R., Supriyadi R. (2019). The growing burden of end-stage renal disease in Indonesia: ten years of the Indonesian renal registry reports. *Kidney International Reports*.

[2] Ernawati D. S., Soebadi B., Radithia D. (2010). Human-leukocyte antigen typing in Javanese patients with recurrent aphthous stomatitis. *Dental Journal (Majalah Kedokteran Gigi)*.

[3] Robson K. J., Ooi J. D., Holdsworth S. R., Rossjohn J., Kitching A. R. (2018). HLA and kidney disease: from associations to mechanisms. *Nature Reviews Nephrology*.

[4] Panigrahi A., Agarwal S. K., Kanga U. (2002). Influence of HLA compatibility on renal graft survival using live unrelated & cadaver donors in India. *The Indian Journal of Medical Research*.

[5] Tuladhar A., Shrestha S., Raut P. P., Bhandari P., Shrestha P. (2013). HLA antigen distribution in renal transplant recipients and donors. *Journal of Nepal Health Research Council*.

[6] Thomas R., Banerjee M. (2005). Original communication HLA-A allele frequency and haplotype distribution in the Dravidian tribal communities of South India. *Indian Journal of Human Genetics*.

[7] Ali M. E., Ahmed M. U., Alam S., Rahman M. H. (2008). HLA-A, -B and -DRB1 allele frequencies in the Bangladeshi population. *Tissue Antigens*.

[8] Pradana K. A., Widjaya M. A., Wahjudi M. (2020). Indonesians human leukocyte antigen (HLA) distributions and correlations with global diseases. *Immunological Investigations*.

[9] Dafalla A. M., Mccloskey D. J., Alemam A. A. (2011). HLA polymorphism in sudanese renal donors. *Saudi Journal of Kidney Diseases and Transplantation: An Official Publication of the Saudi Center for Organ Transplantation, Saudi Arabia*.

[10] Hajjej A., Kaabi H., Sellami M. H. (2006). The contribution of HLA class I and II alleles and haplotypes to the investigation of the evolutionary history of Tunisians. *Tissue Antigens*.

[11] Cao Q., Xie D., Liu J. (2014). HLA polymorphism and susceptibility to end-stage renal disease in cantonese patients awaiting kidney transplantation. *PLoS One*.

[12] Bodis G., Toth V., Schwarting A. (2018). Role of human leukocyte antigens (HLA) in autoimmune diseases. *Rheumatology and Therapy*.

[13] Ravazzi-Gauch C., Maximiliano Bajay M., Cristina Caldas H., Abbud-Filho M. (2016). HLA-A, -B, and -DRB1 allele and haplotype diversity in a cohort of Brazilian renal transplant candidates. *Human Immunology*.

[14] Pan Q., Ma X., Chen H. (2019). A single center study of protective and susceptible HLA alleles and haplotypes with end-stage renal disease in China. *Human Immunology*.

[15] Abdulsalam M., Gony J., Schmid M. (1975). HL-A phenotyping in an Indonesian population. *Tissue Antigens*.

[16] Lowe M., Payton A., Verma A. (2021). Associations between human leukocyte antigens and renal function. *Scientific Reports*.

[17] Png E., Thalamuthu A., Ong R. T. H., Snippe H., Boland G. J., Seielstad M. (2011). A genome-wide association study of hepatitis B vaccine response in an Indonesian population reveals multiple independent risk variants in the HLA region. *Human Molecular Genetics*.

[18] Wasityastuti W., Yano Y., Ratnasari N. (2016). Protective effects of HLA-DPA1/DPB1 variants against hepatitis B virus infection in an Indonesian population. *Infection, Genetics and Evolution*.

[19] Mardian Y., Yano Y., Wasityastuti W. (2017). Genetic polymorphisms of HLA-DP and isolated anti-HBc are important subsets of occult hepatitis B infection in Indonesian blood donors: a case-control study. *Virology Journal*.

[20] Hamdi N. M., Al-Hababi F. H., Eid A. E. (2014). HLA class I and class II associations with ESRD in Saudi Arabian population. *PLoS One*.

[21] Rivera P. S., Márquez G., Cipriani A. M. (2012). HLA class I association with progression to end-stage renal disease in patients from Zulia, Venezuela. *Inmunología*.

[22] Noureen N., Shah F. A., Lisec J. (2020). Revisiting the association between human leukocyte antigen and end-stage renal disease. *PLoS One*.

[23] Karnes J. H., Bastarache L., Shaffer C. M. (2017). Phenome-wide scanning identifies multiple diseases and disease severity phenotypes associated with HLA variants. *Science Translational Medicine*.

